# Daily changes on seasonal ecophysiological responses of the intertidal brown macroalga *Lessonia spicata*: Implications of climate change

**DOI:** 10.3389/fpls.2022.941061

**Published:** 2022-09-28

**Authors:** Paula S. M. Celis-Plá, Andres Trabal, Camilo Navarrete, Macarena Troncoso, Fabiola Moenne, Antonio Zúñiga, Félix L. Figueroa, Claudio A. Sáez

**Affiliations:** ^1^ Laboratory of Aquatic Environmental Research (LACER), Centro de Estudios Avanzados (CEA)/HUB Ambiental UPLA, Universidad de Playa Ancha, Valparaíso, Chile; ^2^ Escuela de Ciencias Agrarias y Veterinarias, Universidad de Viña del Mar, Viña del Mar, Chile; ^3^ Doctorado Interdisciplinario en Ciencias Ambientales, Facultad de Ciencias Naturales y Exactas, Universidad de Playa Ancha, Valparaíso, Chile; ^4^ Ecology Department, Institute of Blue Biotechnology and Development (IBYDA), University of Malaga, Malaga, Spain; ^5^ Departamento de Ciencias del Mar y Biología Aplicada, Facultad de Ciencias, Universidad de Alicante, Alicante, Spain

**Keywords:** *Lessonia spicata*, seasonal changes, photosynthetic activity, productivity, daily cycles

## Abstract

Global climate change is expected to have detrimental effects on coastal ecosystems, with impacts observable at the local and regional levels, depending on factors such as light, temperature, and nutrients. Shifts in dominance between primary producers that can capitalize on carbon availability for photosynthesis will have knock-on effects on marine ecosystems, affecting their ecophysiological responses and biological processes. Here, we study the ecophysiological vulnerability, photoacclimation capacity, and tolerance responses as ecophysiological responses of the intertidal kelp *Lessonia spicata* (Phaeophyceae, Laminariales) during a year through different seasons (autumn, winter, spring, and summer) in the Pacific Ocean (central Chile). Six different daily cycle experiments were carried out within each season. A battery of different biochemical assays associated with antioxidant responses and *in-vivo* chlorophyll *a* fluorescence parameter showed that during spring and summer, there was an increase in photosynthetic capacity in the macroalgae, although their responses varied depending on light and nutrient availability in the course of the year. *Lessonia spicata* showed maximal photosynthesis and a similar photoinhibition pattern in summer compared to the other seasons, and the contents of nitrate and phosphorous in seawater were less in winter. Thus, high irradiance during spring and summer displayed a higher maximal electron transport rate (ETR_max_), irradiance of saturation (Ek), non-photochemical quenching (NPQ_max_), nitrogen and carbon contents, and photoprotector compound levels. Antioxidant activity increased also in summer, the seasonal period with the highest oxidative stress conditions, i.e., the highest level of hydrogen peroxide (H_2_O_2_). In contrast, under low irradiance, i.e., wintertime conditions, *L. spicata* demonstrated lower concentrations of the photosynthetic pigments such as chlorophyll *a* and carotenoids. Our study suggests that macroalgae that are subjected to increased irradiance and water temperature under lower nutrient availability mediated by seasonal changes (expected to worsen under climate change) respond with higher values of productivity, pigment contents, and photoprotective compounds. Thus, our findings strengthen the available evidence to predict that algae in the order Laminariales, specifically *L. spicata* (kelp), could better proliferate, with lower vulnerability and greater acclimation, than other marine species subject to future expected conditions associated with climate change.

## Highlights

− *Lessonia spicata* showed less ecophysiological vulnerability, high photoacclimation under elevated stress conditions, and high irradiance during spring and summer.

− Daily cycle experiments in the seasonal time are a valuable tool for re-evaluating environmental stress factors in *Lessonia spicata.*

− *Lessonia spicata* had a higher activation of photoinhibition mechanisms and productivity in seasonal stress conditions.

## Introduction

Following the increases in atmospheric CO_2_, there has been also a sustained rise in surface solar radiation, being a consequence of the historic destruction of the ozone layer by chlorofluorocarbons (CFCs) and other halogenate ozone-depleting substances (ODS), and these consequences are still observable today (Lu et al., 2017). In this context, solar radiation (irradiance) is a principal factor influencing the primary producers and ecological equilibrium (Pérez-lloréns et al., 2014; Celis-Plá et al., 2016). Indeed, in central Chile, the solar irradiance varies throughout the year, with high levels in summer than in winter. Therefore, the levels of solar irradiance in summer can reach as high as 9,000 kJ m^−2^ photosynthetic active radiation (PAR), which means that the values decrease in other seasons of the year (Zúñiga et al., 2021). These aspects make the area a perfect natural laboratory to undertake cutting-edge research on photobiology and radiation tolerance of photosynthetic organisms (Cabrera et al., 2012; Zúñiga et al., 2021). Although there are some data on solar irradiance levels in the area, there is no information on how the physiology and metabolic performance of photoautotrophs and the ecological balance of coastal waters will be affected in the mid/long term.

Some brown macroalgae are habitat-forming organisms, being the base of trophic networks as primary producers in temperate coastal rocky shores (Buschmann et al., 2014; Celis-Plá et al., 2021). In central and southern Chile, the main intertidal bioengineering species is the kelp *Lessonia spicata* (Ochrophyta) with relevant economic importance in the country (Gómez et al., 2016; Zúñiga et al., 2021). This organism is exposed to a wide range of daily and seasonal changes in PAR and UV, and the latter has been observed to be especially harmful when in excess (Huovinen and Gómez, 2006; Gómez et al., 2016). In brown seaweeds, photosynthetic performance is one of the first physiological aspects affected by exposure to excess solar radiation (Celis-Plá et al., 2021). In this regard, high radiation can result in disruption in electron transport chains in chloroplasts, inducing excess energy transfer to oxygen and the overproduction of reactive oxygen species (ROS) which, subsequently, can lead to oxidative stress and damage (Heinrich et al., 2012). Indeed, the latter can finally cause lipid peroxidation and damage to nucleic acids (Wiencke et al., 2000). In this context, macroalgae are considered vulnerable to high ultraviolet radiation (UVR), when the excess to active the mechanisms of photodamage, photoprotection and photorepair (Murata et al., 2007; Celis-Plá et al., 2016). The concept of vulnerability is associated with photoacclimation capacity, defined as the concentration of photoprotector compounds synthesized under increased irradiance upon light penetration at low tide (Figueroa et al., 2014; Celis-Plá et al., 2017).

Excess solar radiation beyond tolerance can change photosynthetic performance, which has been observed to be a reliable functional indicator of physiological performance in brown macroalgae (Celis-Plá et al., 2021). In this regard, the appropriate evaluation of the functional indicators, such as photosynthetic performance, bio-optic characteristics, effective quantum yield (*ΔF/F_m′_*), maximal quantum yield (*F*_v_/*F*_m_) (*in-vivo* chlorophyll *a* fluorescence associated with Photosystem II), and non-photochemical PSII fluorescence quenching (NPQ), and the study of the electron transport rate (ETR), through its potential extrapolation to the natural environment, to explain the physiological stress conditions that macroalgae are exposed to (Celis-Plá et al., 2016; Celis-Plá et al., 2021; Figueroa et al., 2021).

Polyphenols are photoprotective compounds that protect against higher light irradiance and excessive ROS (Bischof et al., 2006; Celis-Plá et al., 2021). In this regard, the antioxidant properties of polyphenols are related to phenol rings, which serve as electron donors for ROS as peroxy and superoxide anions and hydroxyl radicals (Sathya et al., 2017). Interestingly, it has been observed that polyphenols from brown macroalgae have stronger antioxidant capacities than those of terrestrial plants (Hemat, 2007). Moreover, these compounds can also absorb radiation in the UV spectra (280–400 nm) (Abdala-Díaz et al., 2014). It is also known that high ROS in macroalgal cells is counteracted by the production of other important antioxidant metabolites, such as glutathione and ascorbate, and the activities of antioxidant enzymes including superoxide dismutase (SOD), catalase (CAT), and ascorbate peroxidase (APX), among others (Moenne et al., 2016). All these antioxidant compounds can be either investigated by studying individual components or, alternatively, by estimating total antioxidant capacity (Sáez et al., 2015).

Carotenoids are also important photoprotective compounds in brown macroalgae. Their main role is related to prevent damage to photosynthetic apparatus through different mechanisms; 1) light harvesting and quenching capacity of triplet chlorophyll (Chl); 2) electron donors for ROS reduction; and 3) regulating the rate of thermal energy dissipation in photosynthesis (Demmig-Adams and Adams, 3rd 2006; Raven, 2011; Figueroa et al., 2021). Thermal or heat dissipation is triggered by the trans-thylakoid proton gradient (ΔpH) and zeaxanthin (Zea) synthesis through the xanthophyll cycle (Gilmore, 1997; Figueroa et al., 2021).

There is scarce information on field studies addressing macroalgae responses to natural seasonal and daily changes, with respect to environmental stress factors under climate change conditions, such as temperature, nutrient availability, and solar radiation, and how these are linked to photosynthetic performance, development, and algal productivity. This investigation aims to address the seasonal and daily ecophysiological and metabolic responses in the intertidal brown macroalga *L. spicata*, addressing its vulnerability and photoacclimation capacity, the latter being in front of future scenarios associated with climate change.

## Material and methods

**Figure 1:**
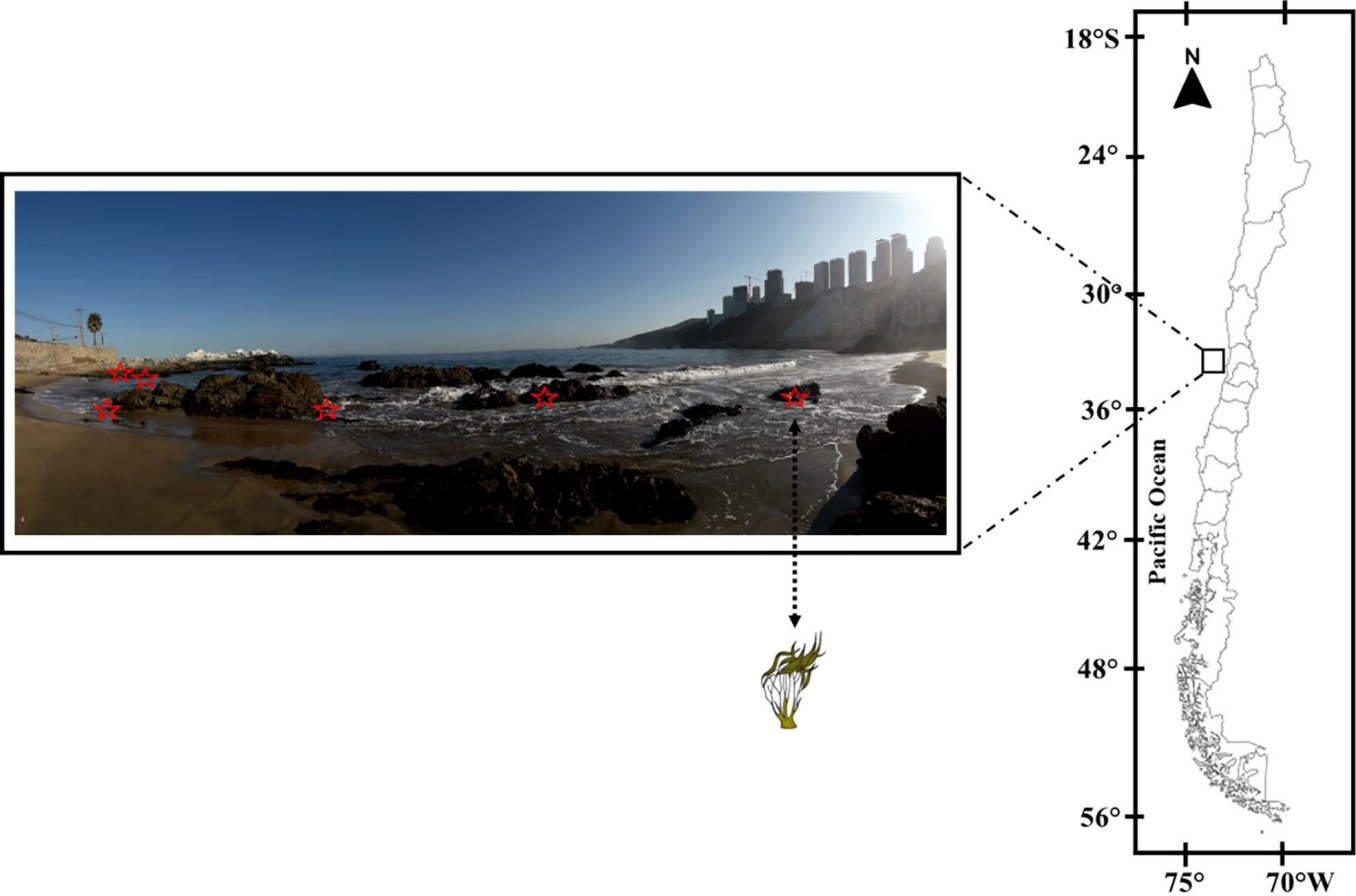
Sampling area at Cochoa Beach in Valparaíso Bay (32°57′19"S; 71°32′52"W) during daily cycle experiments throughout the seasons between 2019 and 2020 with *Lessonia spicata*.

*Lessonia spicata* (Surh) Santelices (Phaeophyceae, Laminariales) were collected in an intertidal area at least 10 m apart along a 70-m transect for 6 days in each season—autumn (4–9 April 2019), winter (23–28 July 2019), spring (18–23 November 2019), and summer (17–22 January 2020)—at the Cochoa Beach, Montemar Marine Protected Area, in Valparaíso Bay (32°57′19"S, 71°32′52"W), Chile (**Figure 1**). For each daily cycle experiment, six adult thalli of *L. spicata* were collected at 10:00, 12:00, 14:00, 16:00, and 18:00 h (local time) from April 2019 to January 2020 and were maintained in trays with 1.5 L of seawater for the ecophysiological measurements. For the biochemical assessments, samples of *L. spicata* were immediately frozen in liquid nitrogen and transported to the Coastal Environmental Research Laboratory, at the Environmental HUB UPLA Center, Universidad de Playa Ancha, and preserved in an ultra-freezer at −80°C for further analyses.

Different environmental parameters were recorded throughout the day and seasons as ground information. Changes in the spectral composition of solar radiation—the irradiance of photosynthetically active radiation or PAR radiation (*λ* = 400–700 nm) and ultraviolet-A (UVA) radiation (*λ* = 315–400 nm)—were quantified through Apogee sensors (Apogee Instruments, USA) with a data logger (HOBO UX120-006M, Onset Computer Corporation, Bourne, MA, USA) according to Quintano et al. (2019); for PAR and UVA, daily doses of irradiance were integrated during the experimental period according to Celis-Plá et al. (2014). Seawater temperature and pH were measured using a multiparameter probe (HI98194, Hanna Instruments, Woonsocket, RI, USA).

### Photosynthetic performance

*In-vivo* chlorophyll *a* fluorescence of photosystem II (PSII) was determined using two fluorometers: 1) MINI-PAM II (red light) and 2) JUNIOR-PAM (blue light) (Walz GmbH, Germany). Basal fluorescence (*F*_o_) and maximum fluorescence (*F*_m′_) were measured under light conditions to obtain the effective quantum yield *(ΔF*/*F*_m′_), where *ΔF*/*F_m′=_ F_m′_ − F_o_/F_m′_*. To determine the maximum quantum yield (*F*_v_/*F*_m_), algal fronds were incubated in a dark chamber with fresh seawater for 15 min before fast light curves were measured, to fully photoreduce all involved reaction centers (Figueroa et al., 2014; Celis-Plá et al., 2014).

The maximum ETR was determined by exposing the tissue for 20 s to 12 irradiances of actinic light in the fast light curve (RLC), using the following formula from Schreiber et al. (1995):


(1) $$ETR\;\left(\mu mol\;electrons\;m^{-2}s^{-1}\right)=△F/F_{m'}\;\times \;E\;\times \;A\;\times \;F_{II}$$

where *ΔF*/*F*_m′_ is the effective quantum yield, *E* is the PAR in each light pulse expressed in μmol m^−2^ s^−1^, *A* is the absorbance of the thalli equivalent to the fraction of the incident irradiance that is absorbed by algae (Figueroa et al., 2003), and *F*_II_ is the portion of chlorophyll *a* related to PSII (400–700 nm), which is 0.8 in brown macroalgae (Figueroa et al., 2014). ETR parameters such as maximum electron transport rate (ETR_max_) and the initial slope of ETR versus irradiance function (*α*_ETR_) as an index of photosynthetic efficiency were calculated from the tangential model by Eilers and Peeters (1988). Saturation irradiance (Ek_ETR_) was calculated from the intersection of ETR_max_ and *α*_ETR_.

Non-photochemical quenching (NPQ) was calculated as proposed by Bilger and Björkman (1990):


(2) $$NPQ\;=Y\left(NPQ\right)/Y\left(NO\right)\;=\;(F_{m}-F_{m'})/F_{m'}$$

The maximum non-photochemical extinction (NPQ_max_), the initial slope of the NPQ function versus irradiance (*α*_NPQ_), and the irradiance saturation of the Ek_NPQ_ were obtained by the tangential model contained in Eilers and Peeters (1988).

### Biochemical variables

Carbon (C) and nitrogen (N) contents were obtained on a dry weight (DW) basis using the elemental analyzer CNHS-932 (LECO, Michigan, USA) (Celis-Plá et al., 2017). All biochemical variables were expressed as mg g^−1^ DW after determining the fresh to dry weight ratio, estimated as 2.84 for *L. spicata*.

The carotenoid and chlorophyll pigment contents for macroalgae were determined to evaluate the capacity for acclimation, photoinhibition, photoprotection, and vulnerability in relation to the different irradiances and abiotic variables.

Total chlorophyll *a* (Chl*a*), chlorophyll *c* (Chl*c*), and carotenoid (Car) contents were extracted from 20 mg of nitrogen-ground fresh tissue with 1.5 ml of 90% acetone. The samples were incubated overnight at 4°C in the dark and then centrifuged at 16,200*g* (NU-C200R, NuWind, Plymouth, MN, USA) for 10 min at 4°C. Finally, Chl*a* and Chl*c* were determined according to Ritchie (2006) (Eqs. 3 and 4), and Car was determined according to Parsons and Strickland (1963) (Eq. 5). These analyses were conducted using a microplate spectrophotometer (SPECTROstar Nano, BMG Labtech, Offenburg, Germany) and calculated as follows:


(3) $$Chla=\;11.47\times (A_{664}-A_{750})\;-\;0.45\times (A_{630}-A_{750})$$

(4) $$Chlc=\;22.679\;\times \;\left(A_{630}-A_{750}\right)-3.404\;\times \;\left(A_{664}-A_{750}\right)$$

(5) $$Carotenoids\;\left(Car\right)\;=\;10\;\times \;\left(A_{480}-A_{750}\right)\;\;\;\;\;\;\;\;\;\;\;$$

The determination and quantification of specific chlorophylls and carotenoids were conducted using high-performance liquid chromatography (HPLC), with modifications to the methods by Celis-Plá et al. (2015); Celis-Plá et al. (2017). Pigments (fucoxanthin, violaxanthin, zeaxanthin, β-carotene, and chlorophyll *a*) were extracted overnight at 4°C from 20 mg fresh weight using 500 µl of DMF, subsequently filtered (0.22-µM nylon filters), and then analyzed using an HPLC Ultimate 3000 (Thermo Fisher Scientific, Waltham, MA, USA). The carotenoid composition was determined using commercial standards (DHI LAB Products, Horsholm, Denmark).

The phenolic compound (PC) contents of *L. spicata* were measured through methanolic extracts. Firstly, 0.25 g of nitrogen-ground fresh tissue samples were added to 2.5 ml of 80% methanol in 15-ml conical tubes, and the mixtures were incubated in the dark overnight at 4°C with vigorous agitation. Then, the whole extracts were centrifuged at 2,253*g* for 30 min at 4°C, and the supernatants were collected to measure PC content using the Folin–Ciocalteu reagent (Merck) and phloroglucinol (Sigma) as standard. Finally, absorbances were read at 760 nm with a microplate reader (Celis-Plá et al., 2016).

Antioxidant activity, determined by the 2,2-diphenyl-1-picrylhydrazyl (DPPH) method, was measured on the same phenolic extracts (according to Blois, 1958). Each extract was added with 150 μl of DPPH, prepared in 90% methanol, and the reaction was completed after 30 min in the dark at ambient temperature (~20°C); the absorbance was read at 517 nm using a microplate reader. The calibration curve made from DPPH was used to calculate the remaining concentration of DPPH in the reaction mixture after incubation (Celis-Plá et al., 2016). Finally, the Trolox compound (6-hydroxy-2,5,7,8-tetramethylchromane-2-carboxylic acid) (0 to 50 µM) was applied as the reference antioxidant. The results obtained were expressed as µmol TEAC (Trolox equivalent antioxidant capacity) g^−1^ DW (Álvarez-Gómez et al., 2017).

The concentrations of H_2_O_2_ were quantified using the colorimetric method described by Sáez et al. (2015). One hundred milligrams of the samples were macerated in liquid nitrogen, acidified, and lysed with 100 µl of 10% trichloroacetic acid (TCA), 100 µl of 10 mM potassium phosphate buffer (pH 7.0), 100 µl of FAPRB lysis buffer (Favorgen, Wien, Austria), and 500 µl of 1 M KI. Then, the samples were vortexed for 10 min at room temperature in the dark and centrifuged at 12,000*g* for 15 min at 4°C. Three hundred microliters of the supernatant was measured at 350 nm using a microplate reader. Controls were performed for each sample by adding 500 µl of ultrapure water instead of 500 µl of KI. Thirty percent of H_2_O_2_ (Merck) was used for the standard curve with concentrations from 0 to 20 µg ml^−1^.

As a proxy for lipid peroxidation, the levels of thiobarbituric acid-reactive substance (TBARS) were measured according to Sáez et al. (2015). One hundred milligrams of the samples previously macerated in liquid nitrogen were lysed with 300 µl of 10% TCA, adding four glass beads (3 mm) and vortexed for 15 min at room temperature. Then, the samples were centrifuged at 17,800*g* for 15 min at 4°C, and 200 µl of the supernatant was mixed with 200 µl of 0.5% thiobarbituric acid (TBA) diluted in 10% TCA and then incubated at 95°C for 45 min. Finally, the samples were cooled to room temperature and measured at 532 nm using a microplate reader. Controls were performed for each sample by adding 200 µl of 10% TCA instead of 200 µl of 0.5% TBA. The 1,1,3,3-tetramethoxypropane standard was used to perform the standard curve with concentrations from 0 to 25 µM. Reduced glutathione (GSH) was determined using the enzymatic recycling method as described for brown macroalgae in Sáez et al. (2015) but optimized for *L. spicata*. Finally, reduced ascorbate (ASC) was measured according to the ferric reducing/antioxidant power assay (Sáez et al., 2015) with modifications.

### Statistical analysis

The interactive effects of the *in-situ* design on the ecophysiological responses of *L. spicata* were assessed using analysis of variance (ANOVA; Underwood, 1997). For the physiological responses, three fixed factors were considered: seasonal time with four levels (autumn, winter, spring, and summer), time with six levels (day 1, day 2, day 3, day 4, day 5, and day 6), and hours with five levels (10:00, 12:00, 14:00, 16:00, and 18:00 h). For biochemical responses, ANOVA was applied with three fixed factors: seasonal time with four levels (autumn, winter, spring, and summer), time with six levels (day 1, day 2, day 3, day 4, day 5, and day 6), and hours with three levels (10:00, 14:00, and 18:00 h). Student–Newman–Keuls tests (SNK) were performed on significant ANOVA interactions. Homogeneity of variance was evaluated using the Cochran tests and by visual inspection of the residuals. All data conformed to the homogeneity of variance (Underwood, 1997). Analyses were performed using SPSS v.21 (IBM, USA).

A principal coordinates analysis (PCA), redundancy analysis (dbRDA), and distance-based linear models (DistLM) were performed using PERMANOVA+ for the PRIMER 6 package (Anderson et al., 2008). PCA was calculated based on the Euclidean distance, multivariate ordination was used to investigate the contribution of ecophysiological responses to variance, and the ordination plot was also studied. Each of the variables was represented by an arrow in the ordination plot pointing to the samples that showed the highest amount of each compound. Each replicate represented the content of all variables calculated from the six thalli taken at one collected sample for each daily cycle and seasonal time. The dbRDA was performed as a multivariate approach to determine a general variation of patterns between all ecophysiological and environmental variables, such as solar irradiance, temperature, pH, and nutrients. Before the dbRDA analyses, a preliminary distance-based linear model with the AIC selection criterion was carried out for each site (Anderson et al., 2008).

## Results

### Environmental variables

During winter, the average daily values of nitrate ranged from 21.21 to 18.18 µM, the seawater temperature ranged from 12.34°C to 14.27°C, the irradiance of PAR ranged from 502 to 928.22 µmol m^−2^ s^−1^, and UVA ranged from 1.20 to 5.95 W m^−2^ (**Table 1**). In summer, the average daily values of nitrate ranged from 3.86 to 8.05 µM, seawater temperature from 14.27°C to 17.83°C, the irradiance of PAR from 182.66 to 1,206 µmol m^−2^ s^−1^, and UVA from 1.93 to 10.10 W m^−2^ (**Table 1**).

**Table 1:** The abiotic parameters nitrate (NO_3_^−^ expressed in µM), phosphate (PO_4_^3−^ expressed in µM), temperature (expressed in °C), pH (expressed in a range from 0 to 14), photosynthetically active radiation (PAR expressed in µmol photons m^−2^ s^−1^), and ultraviolet-A radiation (UVA expressed in W m^−2^) at Cochoa Beach in Valparaíso Bay during daily cycle experiments with *Lessonia spicata* in autumn, winter, spring, and summer, between 2019 and 2020.

Seasons	Daily cycle	Nitrate (µM)	Phosphate (µM)	Temperature (°C)	pH	PAR (µmol photons m^−2^ s^−1^)	UVA (W m^−2^)
Autumn	10:00	3.99 ± 0.26	0.98 ± 0.05	12.29 ± 0.01	7.84 ± 0.01	381.04 ± 100.42	4.07 ± 0.40
	14:00	3.41 ± 0.27	0.91 ± 0.03	12.29 ± 0.01	7.85 ± 0.01	986.34 ± 261.10	12.11 ± 1.67
	18:00	3.31 ± 0.28	0.97 ± 0.07	12.29 ± 0.01	7.88 ± 2.15	177.45 ± 53.98	3.30 ± 0.27
Winter	10:00	21.21 ± 1.10	1.98 ± 0.06	11.71 ± 0.03	7.87 ± 0.01	502.31 ± 68.38	4.13 ± 0.21
	14:00	19.92 ± 0.63	1.80 ± 0.05	12.42 ± 0.06	7.95 ± 0.01	928.22 ± 77.17	5.95 ± 0.19
	18:00	18.18 ± 1.10	1.84 ± 0.06	12.34 ± 0.07	8.00 ± 0.01	9.05 ± 1.15	1.20 ± 0.02
Spring	10:00	6.47 ± 0.88	1.26 ± 0.07	14.15 ± 0.24	7.91 ± 0.03	188.83 ± 23.14	2.75 ± 0.15
	14:00	5.80 ± 0.74	1.16 ± 0.06	15.45 ± 0.30	8.12 ± 0.02	1,062.18 ± 396.69	14.83 ± 3.61
	18:00	5.65 ± 0.75	1.19 ± 0.06	15.66 ± 0.01	8.17 ± 0.01	561.16 ± 75.08	2.35 ± 0.06
Summer	10:00	8.05 ± 0.96	1.44 ± 0.11	14.27 ± 0.01	7.81 ± 0.01	182.66 ± 27.42	1.93 ± 0.11
	14:00	5.53 ± 1.33	1.28 ± 0.09	16.77 ± 0.02	8.16 ± 0.01	1,206.00 ± 180.42	10.10 ± 2.17
	18:00	3.86 ± 0.81	1.28 ± 0.07	17.83 ± 0.02	8.17 ± 0.01	247.33 ± 28.19	1.98 ± 0.04

### Seasonal multivariable analyses

**Figure 2:**
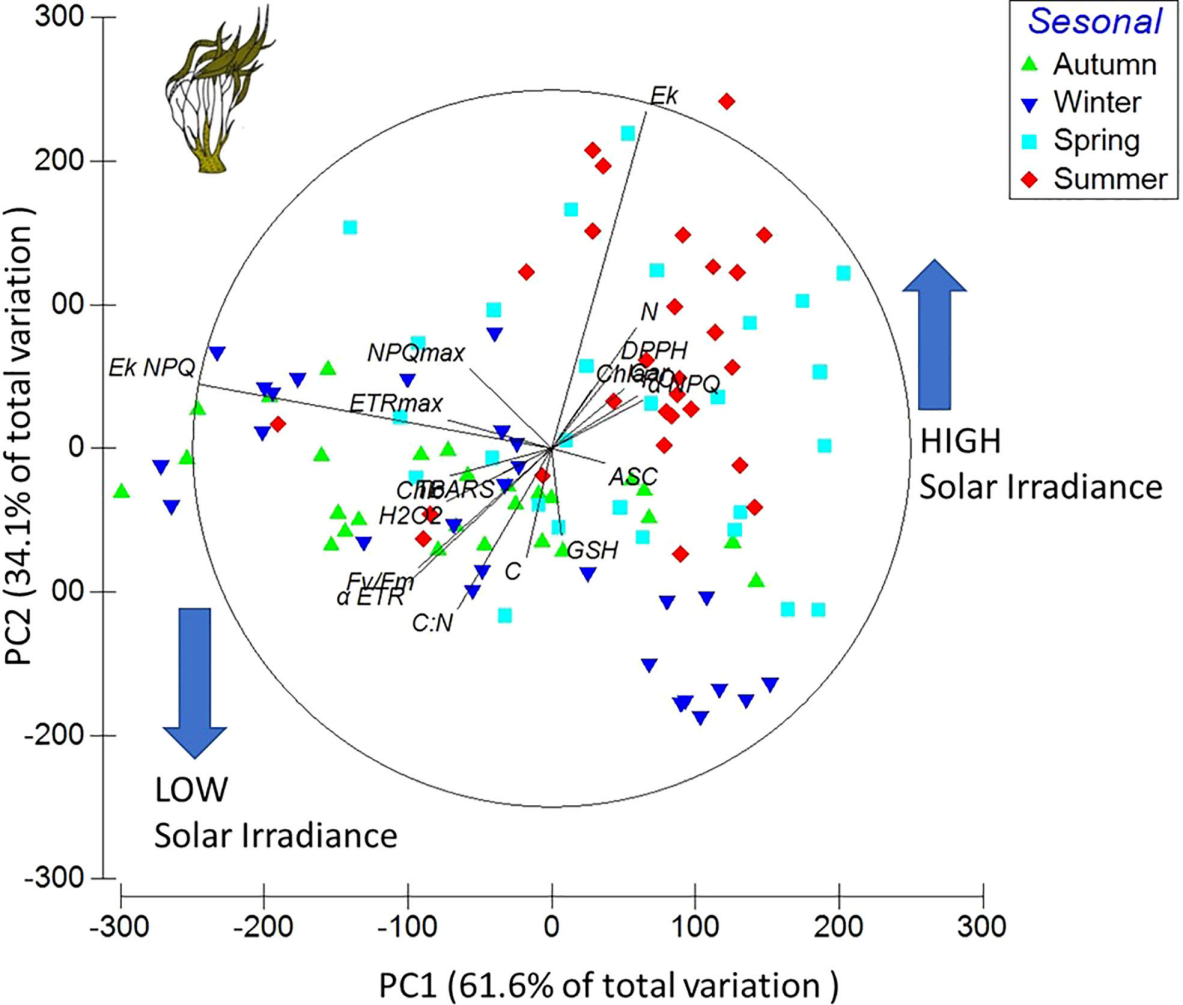
Principal components diagram based on 19 variables and treatment data from *Lessonia spicata* experiments. The ecophysiological variables considered were ETR_max_, *α*_ETR_, Ek, *F*_v_/*F*_m_, *α*_NPQ_, NPQ_max_, Ek_NPQ_, C, N, C:N, Chl*a*, Chl*c*, Car, PC, DPPH, H_2_O_2_, TBARS, GSH, and ASC.

The first axis of the PC analysis explained 61.6% of the total variation with a positive correlation with GSH, ASC, *α*_NPQ_, DPPH, N, Chl*a*, and Ek (ecophysiological variables), mainly occurring when the solar irradiance was increased, i.e., in summer and spring. The second axis explained 34.1% of the total variation and had a positive correlation with *F*_v_/*F*_m_, ETR_max_, NPQ_max_, Ek_NPQ_, *α*_ETR_, Ek_ETR_, Chl*b*, H_2_O_2_, TBARS, C:N, and C (ecophysiological variables), when the solar irradiance was lower, i.e., in winter and autumn. The combination of the first two axes explained 95.7% of the variation for these variables (**Figure 2**).

**Figure 3:**
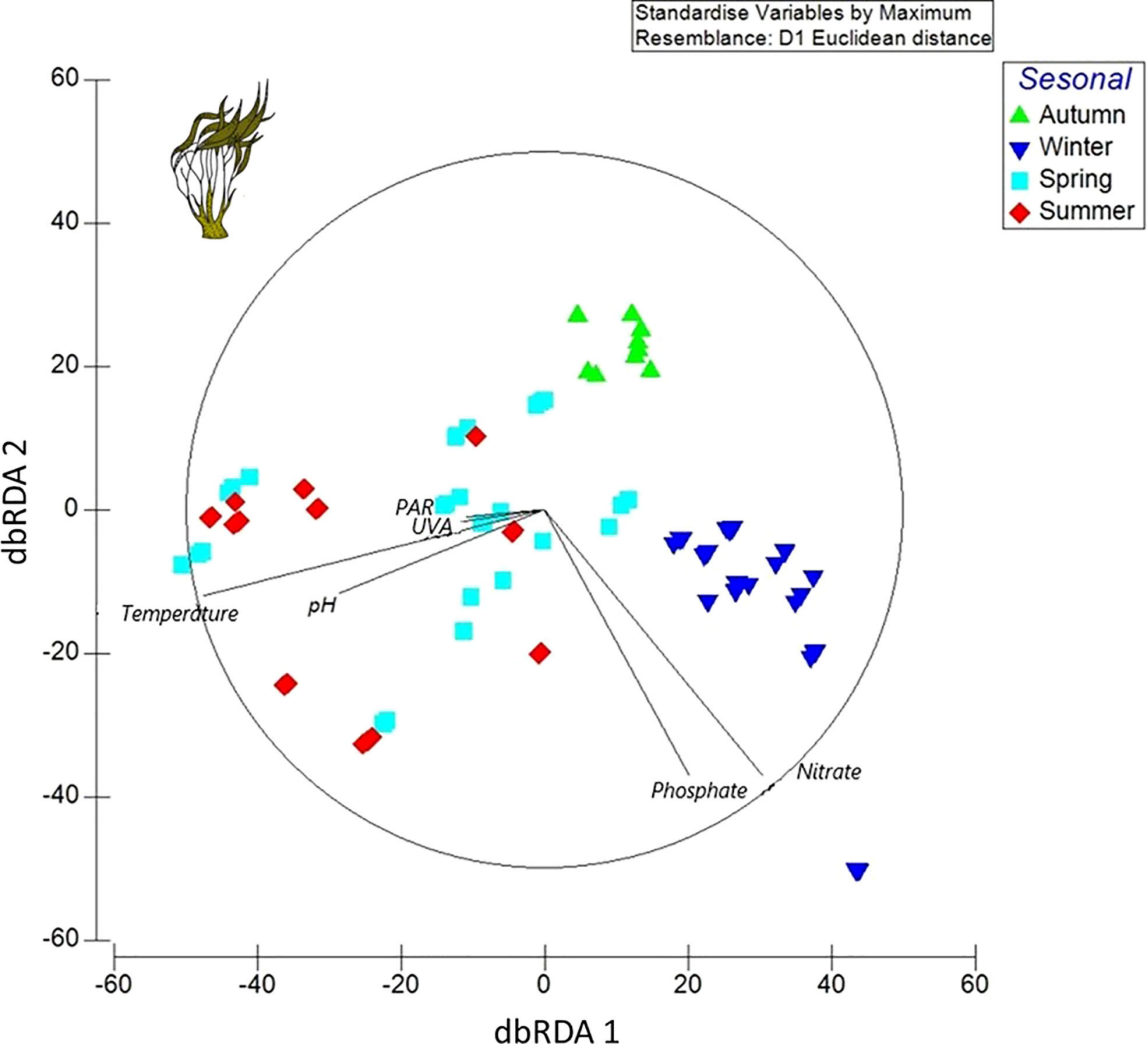
Redundancy analysis (dbRDA diagram) of *Lessonia spicata* throughout the seasons considering ecophysiological variables related to abiotic parameters: PAR (photosynthetically active radiation), UVA (ultraviolet-A), temperature, pH, and NO_3_^−^ (nitrate); with respect to seasons: autumn (green triangle), winter (blue invert triangle), spring (light blue square), and summer (red diamond).

According to the results of the distance-based linear models, which were performed through redundancy analysis, the best spatial arrangement of the samples was described by the distance-based multivariate linear model analysis (DistLM), with the AIC selection criterion. In the preliminary DistLM analysis for autumn, winter, spring, and summer, the ecophysiological variables and all the environmental variables studied explained the best spatial arrangement for all samples with the six categorical environmental variables, in the following order: 1) nitrate, 2) phosphate, 3) temperature, 4) pH, 5) PAR, and 6) UVA. The dbRDA ordination explained 54.25% (dbRDA1) and 26.95% (dbRDA2) of the variation out of the fitted model, in relation to the ecophysiological and biochemical data, and displayed a season separation based on the abiotic factor response. Thus, the nutrient (nitrate and phosphate) categorical factor demonstrated a significant covariation for the samples in winter and autumn; in consequence, these variables increased with decreasing irradiance. In contrast, ecophysiological variables in the spring and summer periods displayed a clear increase and were better explained by temperature, pH, PAR, and UVA (**Figure 3**).

### Ecophysiological responses

**Figure 4:**
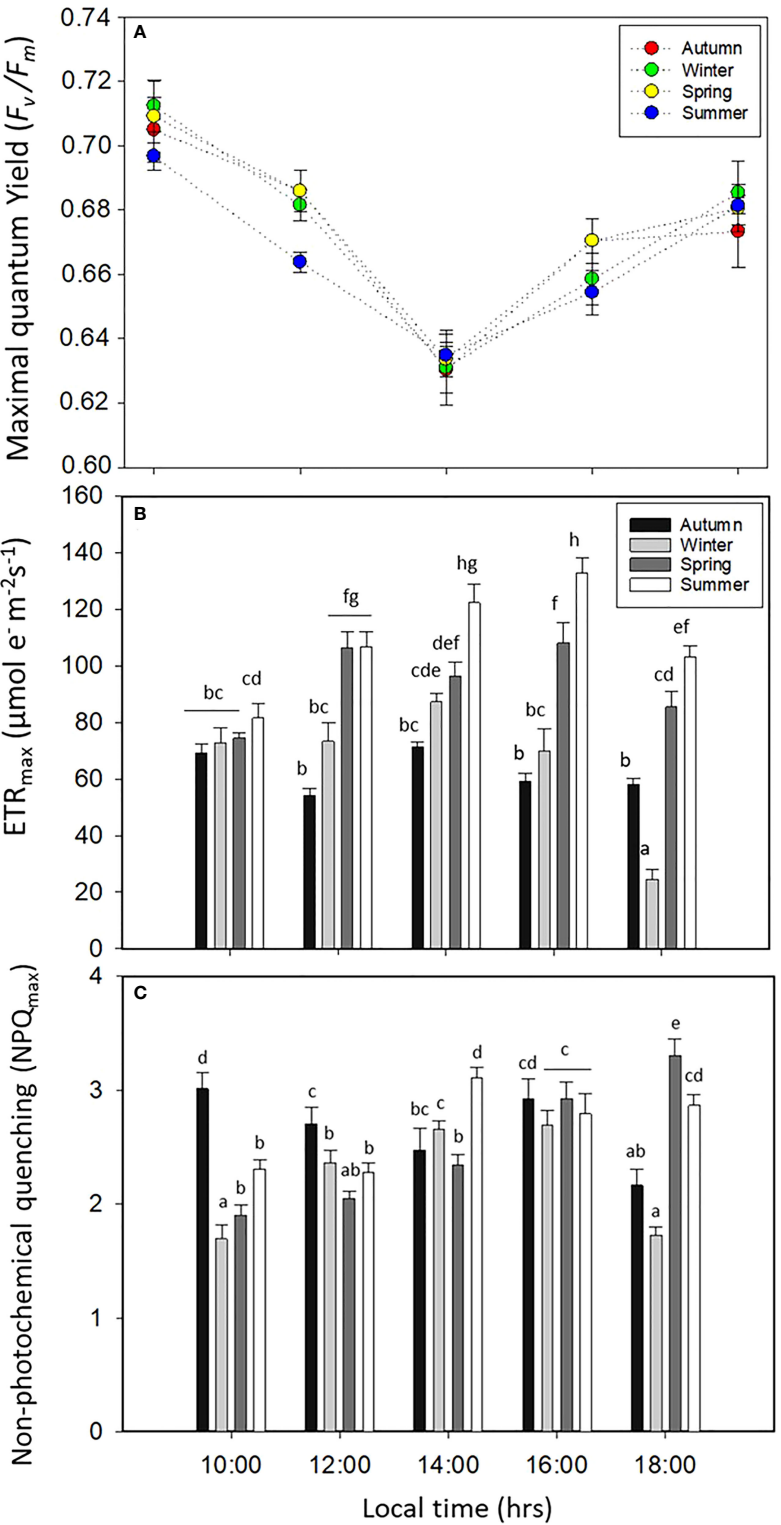
**(A)** Maximal quantum yield (*F*_v_/*F*_m_), **(B)** maximal electron transport rate (ETR_max_), and **(C)** non-photochemical quenching (NPQ_max_) in *Lessonia spicata* during the daily cycle experiments in autumn, winter, spring, and summer between 2019 and 2020 in Valparaíso Bay. Lower - case letters denote significant after the SNK test.

*F*_v_/*F*_m_, ETR_max_, and NPQ_max_ had significant differences for all factors (*p* < 0.05, **Figure 4**, **Table S1**). *F*_v_/*F*_m_ decreased significantly (*p* < 0.05, **Figure 4A**, **Table S1**) at midday for all seasons, with percentages of the decay of about 8.5% for winter, 7.8% for spring, 8% for autumn, and 5.5% for summer, in relation to the morning time (**Figure 4A**). ETR_max_ increased in summer and spring with values of about 100–120 µmol m^−2^ s^−1^; in contrast, the productivity or ETR_max_ was lower in winter (**Figure 4B**). NPQ_max_ increased in spring and summer (**Figure 4C**), and the ratio between ETR_max_/NPQ_max_ (productivity:photoprotection) was higher in spring and summer (**Figures 4B, C**).

**Figure 5:**
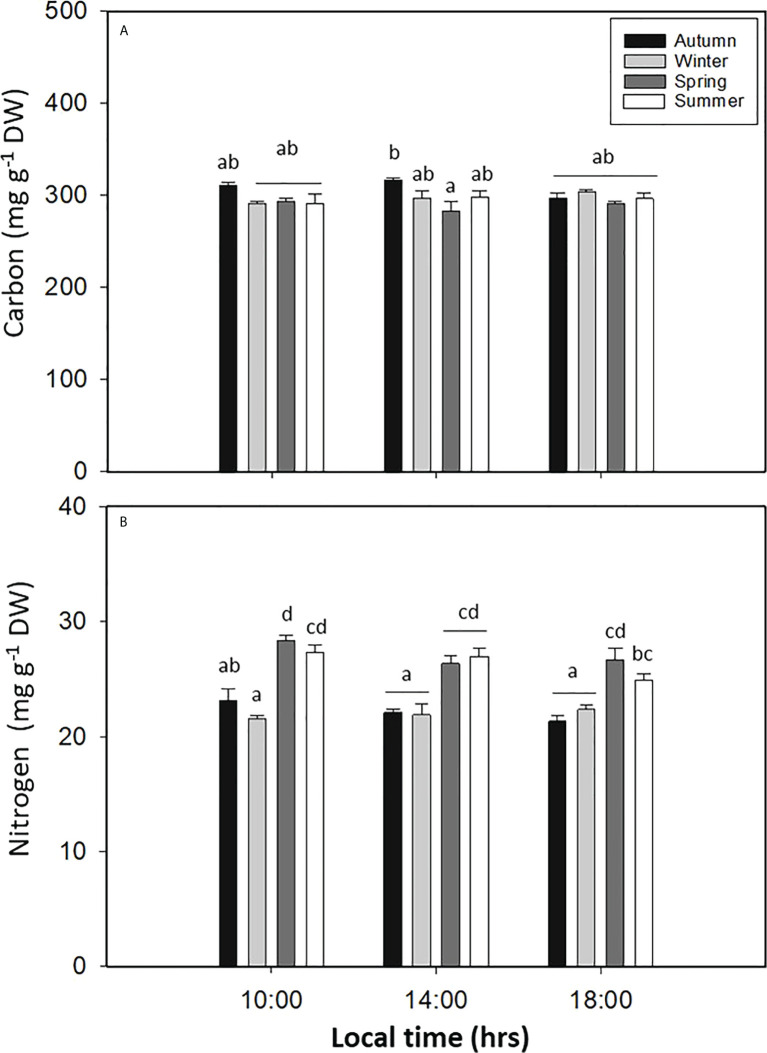
**(A)** Total carbon (mg g^−1^ DW) and **(B)** total nitrogen contents in *Lessonia spicata* under daily cycle experiments in autumn, winter, spring, and summer between 2019 and 2020 in Valparaíso Bay. Lower - case letters denote significant after the SNK test.

The carbon content in *L. spicata* showed significant differences for all seasons (*p* < 0.05, **Figure 5A**, **Table S1**). In autumn, C was recorded with values as high as 310.71 ± 3.31 mg g^−1^ DW at 10:00 h; in contrast, in spring, C had the lowest value of 283.29 ± 10.32 mg g^−1^ DW at 14:00 h (**Figure 5A**). The nitrogen content in *L. spicata* demonstrated significant differences for all factors (*p* < 0.05, **Figure 5B**, **Table S1**). Thus, N had the highest value during spring with 28.33 ± 0.43 mg g^−1^ DW at 10:00 h, and it had the lowest levels in winter with 21.60 ± 0.27 mg g^−1^ DW (**Figure 5B**).

**Figure 6:**
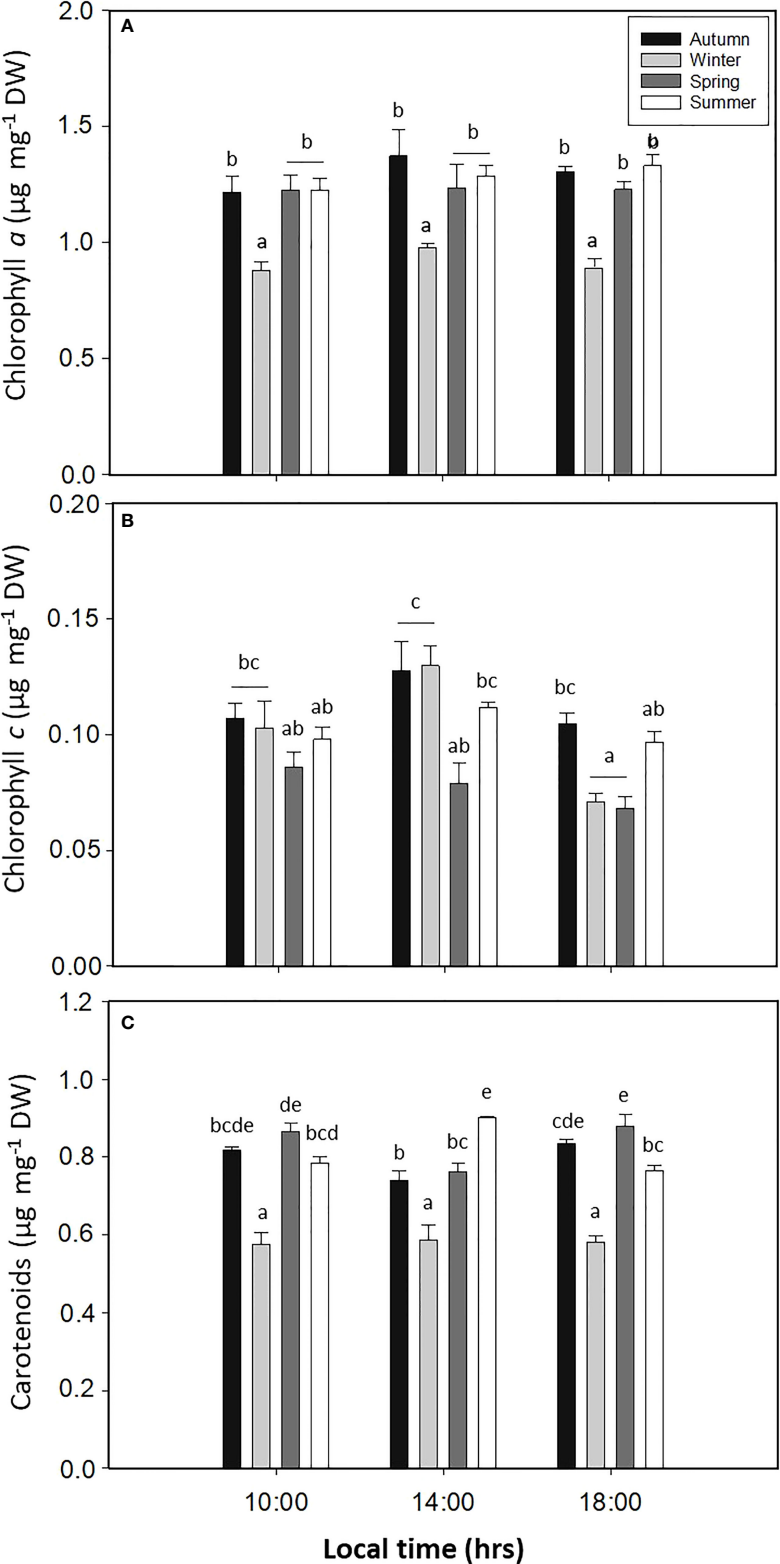
**(A)** Chlorophyll *a*, **(B)** chlorophyll *c*, and **(C)** total carotenoids in *Lessonia spicata* under daily cycle experiments in autumn, winter, spring, and summer between 2019 and 2020 in Valparaíso Bay. Lower - case letters denote significant after the SNK test.

The Chl*a* content had a significant difference for the season factor (*p* < 0.05, **Figure 6A**, **Table S1**), with lower values during spring (**Figure 6A**). Meanwhile, the content of Chl*c* had significant differences for all factors (*p* < 0.05, **Figure 6B**, **Table S1**), with higher levels for autumn and winter, with 0.13 ± 0.01 and 0.13 ± 0.01 µg mg^−1^ DW, respectively (**Figure 6B**). Finally, the Car content in *L. spicata* demonstrated significant differences for all factors (*p* < 0.05, **Figure 6C**, **Table S1**), with higher values of 0.88 ± 0.03 µg mg^−1^ DW in summer at 14:00 h and 0.87 ± 0.02 µg mg^−1^ DW in spring at 18:00 h (**Figure 6C**).

**Figure 7:**
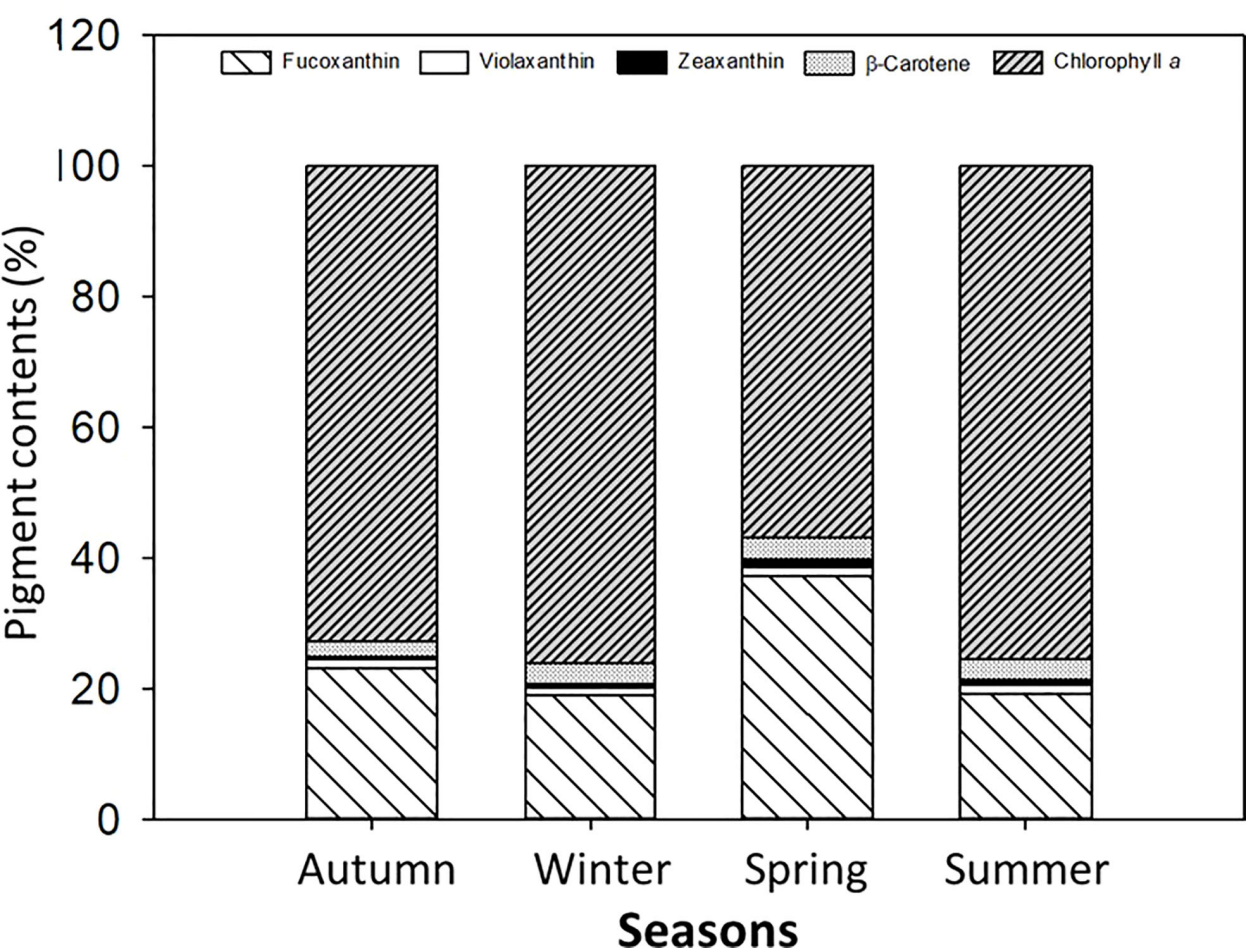
Pigment contents (%) identified in *Lessonia spicata*. According to the legends, fucoxanthin, violaxanthin, zeaxanthin, β-carotene, and chlorophyll *a* were measured in autumn, winter, spring, and summer between 2019 and 2020 in Valparaíso Bay. Lower - case letters denote significant after the SNK test.

The single carotenoid and pigment contents (%), namely, fucoxanthin (Fuco), violaxanthin (Viola), Zea, β-carotene (β-caro), and Chl*a*, were identified in the macroalga *L. spicata* in different proportions (**Figure 7**). In this regard, Fuco had values of about 20%–40% in spring, and Chl*a* had about 75% in autumn, winter, and summer.

**Figure 8:**
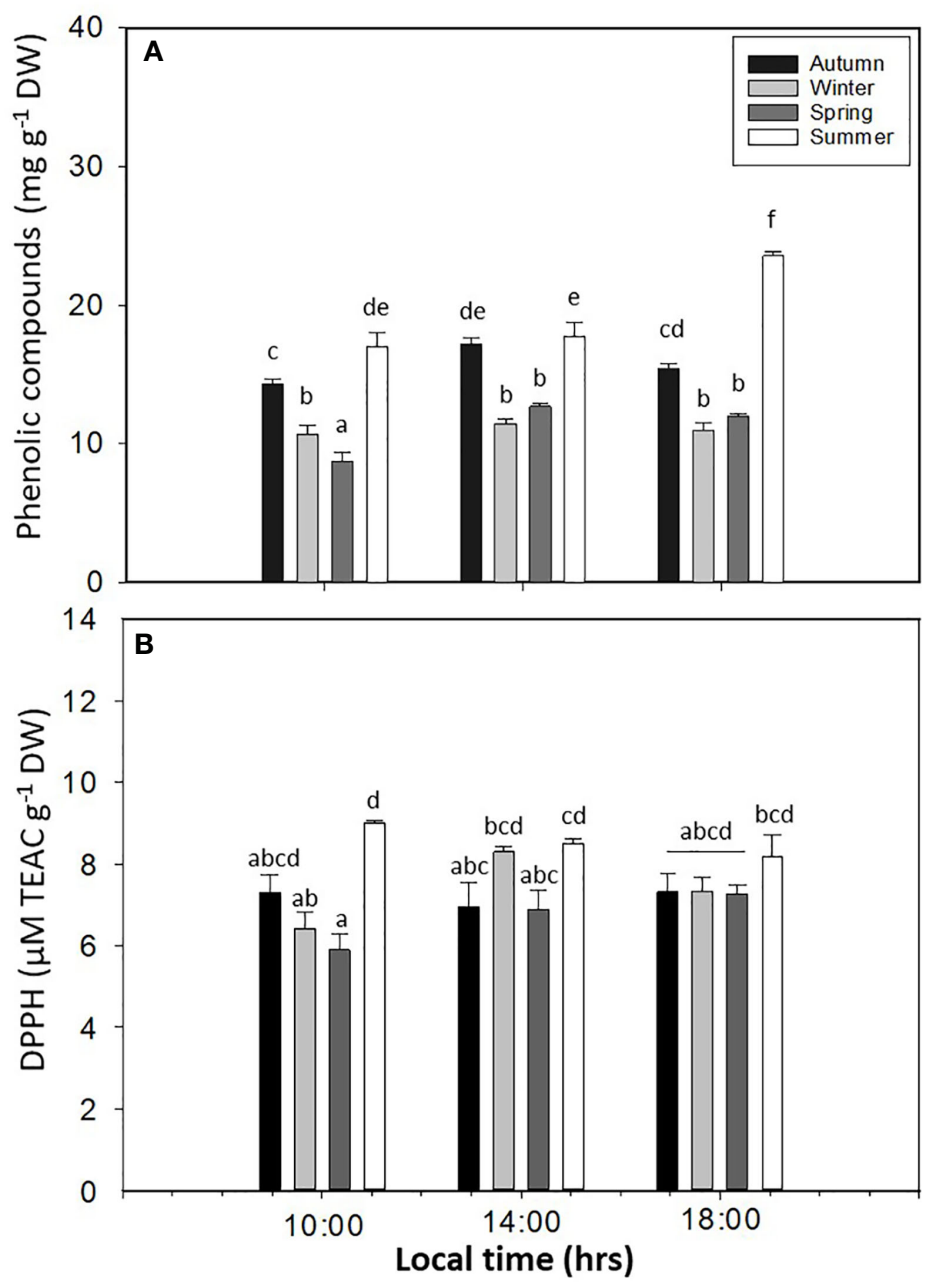
**(A)** Phenolic compounds and **(B)** DPPH antioxidant method in *Lessonia spicata* under daily cycle experiments in autumn, winter, spring, and summer between 2019 and 2020 in Valparaíso Bay. Lower - case letters denote significant after the SNK test.

**Figure 9:**
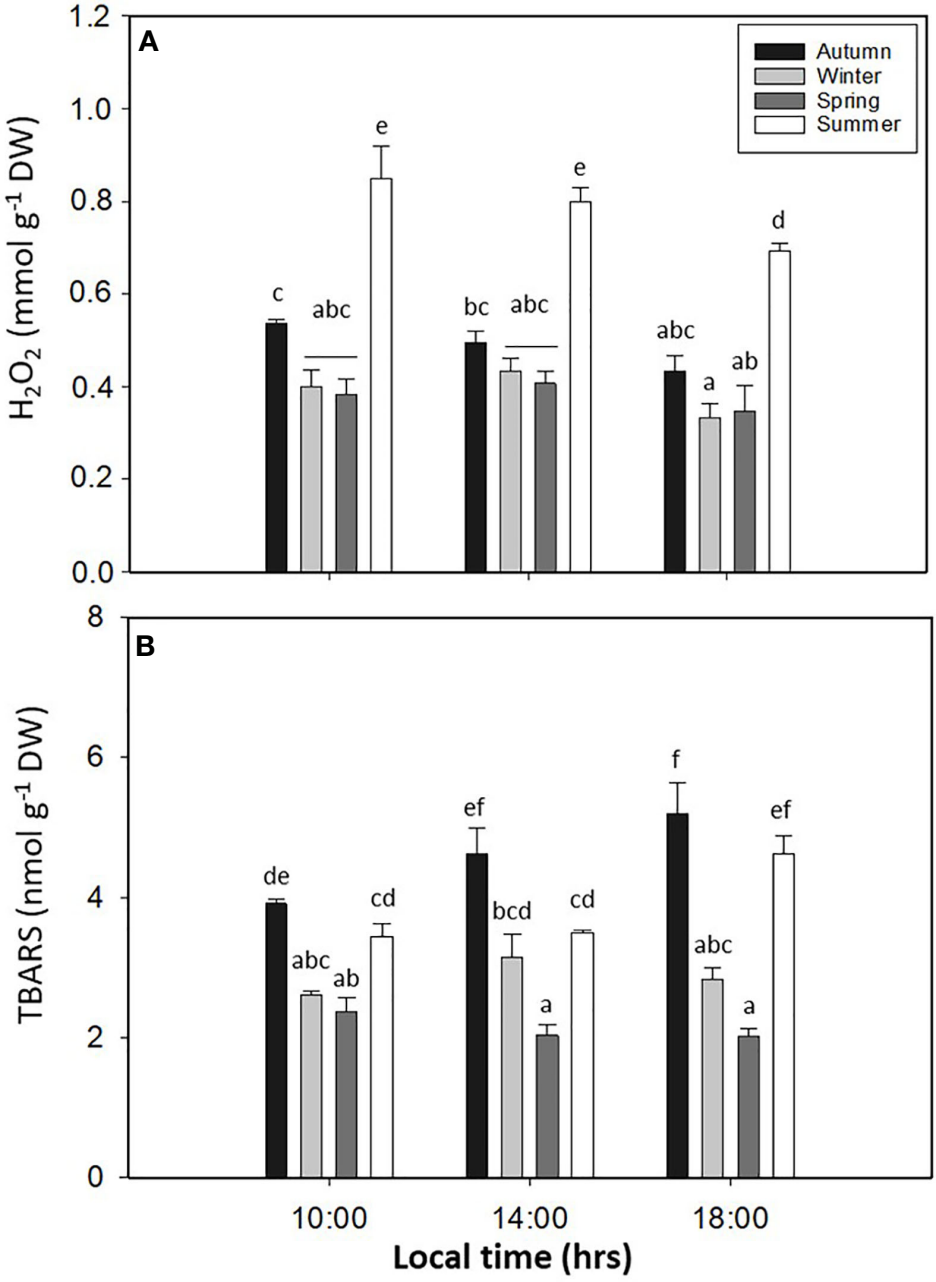
**(A)** Hydrogen peroxide (H_2_O_2_) and **(B)** thiobarbituric acid-reactive substance (TBARS) accumulation in *Lessonia spicata* exposed to daily cycle experiments in autumn, winter, spring, and summer between 2019 and 2020 in Valparaíso Bay. Lower - case letters denote significant after the SNK test.

PC showed significant differences in *L. spicata* for all factors (*p* < 0.05, **Figure 8A**, **Table S1**). The highest values of PC were registered in summer at 14:00 and 18:00 h, with 17.06 ± 0.98–23.59 ± 0.28 mg g^−1^ DW, respectively. Antioxidant activity had significant differences for all factors (*p* < 0.05, **Figure 8B**, **Table S1**). Similar to PC, DPPH had higher values in summer, with a positive correlation between both (0.98; *p* < 0.0016) biochemical variables (**Figure 8B**). The levels of H_2_O_2_ showed significant differences for all factors (*p* < 0.05, **Figure 9A**, **Table S1**). H_2_O_2_ had the highest values in summer than in the other seasons (**Figure 9A**). Finally, the levels of TBARS displayed significant differences for all factors (*p* < 0.05, **Figure 9B**, **Table S1**). TBARS increased in autumn and summer, with 5.20 ± 0.45 mmol g^−1^ DW in autumn at 18:00 h and 4.62 ± 0.26 mmol g^−1^ DW in summer at 18:00 h (**Figure 9B**).

## Discussion

Here, we studied the ecophysiological vulnerability, photoacclimation capacity, and stress tolerance responses of the brown macroalga *L. spicata* in daily seasonal changes in central Chile, under climate change scenarios, with observable impacts depending on the environmental stress factors, such as solar irradiance, temperature, and nutrients. These impacts can have consequences on different parameters for this alga, such as biogeographical distribution, altering the distribution limits (Figueroa et al., 2019), and biomass production (Buschmann et al., 2014), among others. We have proven these based on ecophysiological responses and biological processes, such as photoprotection and antioxidant capacity, which can be extrapolated to the seasonal vulnerability of the species. In this way, a battery of different biochemical assays associated with antioxidant responses and *in-vivo* chlorophyll *a* fluorescence parameters showed that in spring and summer (under increased irradiance) *L. spicata* had greater activation of photoinhibition mechanisms and productivity, displaying lower *F*_v_/*F*_m_ and higher ETR_max_, Ek, NPQ_max_, nitrogen and carbon contents, photoprotector compound levels, pigment contents, antioxidant activity, and H_2_O_2_ concentrations.

The results may suggest that solar irradiance, nutrients, and temperature acting alone or in synergy affected highly the ecophysiology and metabolic process of the macroalga *L. spicata* (Raven, 2011; Hanelt and Figueroa, 2012; Raven and Hurd, 2012; Falkenberg et al., 2013). For instance, the interactions among such factors may determine the success of the macroalgae species and their primary productivity (Celis-Plá et al., 2017). In this context, we observed that temperature and solar irradiance mediated the significant increase in productivity, photoprotective mechanism, and antioxidant activity, among others, in spring and summer. In contrast, we found that *F*_v_/*F*_m_ or photoinhibition of the photosynthetic activity was higher with a decay of about 5.5% in the central hours of the day (14:00 h local time), in relation to morning time, under greater solar irradiance, with about 1,200 µmol photons m^−2^ s^−1^ values at midday in daily cycles in summer; as expected, photoinhibition was lower with a decay of about 8.5% at decreased irradiance and temperature conditions in winter (Adams et al., 2006; Raven, 2011; Figueroa et al., 2021). In contrast, Varela et al,. (2018) showed a reduction pattern of rETR_max_ toward late spring and summer with increased irradiance, temperature, and/or nitrogen availability in *Macrocystis pyrifera* in the south of Chile. In the same way, Cabello-Pasini et al. (2000) observed photoinhibition of photosynthesis in the same kelp specie during solar noon compared to values in the morning and afternoon. In this study, the productivity or ETR_max_ increased under high solar irradiance and temperature in spring and summer when nutrients are less available. In contrast, during winter, high nutrients in the seawater, combined with lower solar irradiance and temperatures, may explain the low ETR_max_ value. This suggests that species that naturally grow at lower depths might be less susceptible to excessive light when cultured in shallow waters.

Contrary to the findings of this study, Celis-Plá et al. (2015) recorded that ETR_max_ in the brown macroalga *Cystoseira tamariscifolia* was reduced under high nitrate levels and irradiance. Moreover, this macroalga grown under increased irradiance, with an average daily solar radiation of ca. 10,165 kJ m^−2^ of PAR, 1,051 kJ m^−2^ of UVA, and 57.5 kJ m^−2^ of UVB, in summer had a greater antioxidant response and lower vulnerability than in winter, with 4,000 kJ m^−2^ of PAR, 400 kJ m^−2^ of UVA, and 15 kJ m^−2^ of UVB (Celis-Plá et al., 2016). Similarly, Figueroa et al. (2014) showed higher antioxidant activity under high PAB (PAR + UV) conditions in *C. tamariscifolia* under laboratory conditions. Furthermore, Celis-Plá et al. (2016) showed that the highest photosynthetic capacity, measured through ETR_max_, occurred during springtime when the concentrations of nutrients were higher, with NO_3_^−^ of about 1.59 µM, NH_4_^+^ of about 0.35 µM, and PO_4_^3−^ of about 0.15 µM. Therefore, the results of the current study may be explained by the acclimation capacity of *L. spicata* to withstand the water stress conditions in central Chile.

An important photoprotective mechanism available to macroalgae is the ability to dissipate excess thermal energy (Adams et al., 2006; Celis-Plá et al., 2016; Figueroa et al., 2021). The NPQ of the PSII is delivered through the xanthophyll cycle, in the synthesis of Zea and ΔpH (Gilmore, 1997; Figueroa et al., 2019; Figueroa et al., 2021). We used NPQ_max_ as an indicator of photoprotective capacity and dissipation efficiency; in this regard, we observed high values in spring and summer, confirming a photoprotective role. *Lessonia spicata* displayed higher NPQ_max_ in autumn and spring, in contrast to that shown by Celis-Plá et al. (2015), with higher NPQ_max_ in *C. tamariscifolia* under treatments with reduced solar irradiance and nutrient enrichment. In this study, we observed that phenolic compounds and antioxidant activity were mainly activated with high solar irradiance and temperature and also with a high internal nitrogen content. In this context, we found a higher than internal nitrogen content of about 20–25 mg g^−1^ DW, accompanied by an antioxidant activity of about 8–10 mg g^−1^ DW in summer. These results can be explained by the high photoacclimation and photoprotection capacity of *L. spicata* under the stressful conditions of summer, which is explained by the high internal nitrogen content to produce high phenolic compounds with increased antioxidant activity.

In *L. spicata* (ex *Lessonia nigrescens*) in the coastal Pacific Ocean, Gómez et al. (2016) found that the synthesis of phlorotannins of 10–25 mg g^−1^ DW was induced by greater UV radiation in summer, in response to the interplay between solar radiation (UV) and the morphofunctional patterns intrinsic of this macroalga. However, a higher value of the phenolics compounds c.a. 13 - 14 mg g^-1^ DW, under high solar irradiance (Zúñiga et al., 2021). In this context, the results of this study suggest the activation of the photoprotective mechanism (NPQ_max_, phenolics, antioxidants) under elevated solar irradiance combined with elevated temperature. In this regard, some similar macroalgae species, such as *M. pyrifera* (Buschmann et al., 2014; Varela et al., 2018; Celis-Plá et al., 2021; Palacios et al., 2021) and *L. spicata* (Gómez et al., 2016; Zúñiga et al., 2021) in the Southern Hemisphere and *C. tamariscifolia* (Figueroa et al., 2014; Celis-Plá et al., 2016; Celis-Plá et al., 2017) in the Northern Hemisphere, will have a higher capacity of photoacclimation through the photoprotective mechanism in future stress conditions.

## Conclusions

Our study demonstrated that the specie *L. spicata* has been subjected to increased stress conditions mediated by seasonal changes, and these patterns are expected to worsen in future climate scenarios; these trends follow the higher values of productivity, pigment contents, and photoprotective compounds observed. However, our findings strengthen the available evidence to predict that algae in the order Laminariales, specifically *L. spicata* (brown macroalgae), could better proliferate, with lower vulnerability and greater acclimatization, than other marine species. We demonstrated that elevated solar irradiance could enhance productivity and photosynthetic performance.

## Data availability statement

The original contributions presented in the study are included in the article/**Supplementary Material**. Further inquiries can be directed to the corresponding author.

## Author contributions

PC-P and CS: investigation, conceptualization, formal analysis, and writing—original draft and review and editing. FF: conceptualization, formal analysis, statistical analysis, and writing—review and editing. AT, CN and MT: experimental research, conceptualization, formal analysis, and statistical analysis. FM and AZ: experimental research. All authors contributed to the article and approved the submitted version.

## Funding

Financial and logistical support was granted by the project of Fondo Nacional de Desarrollo Científico y Tecnológico, Chile through grant Project FONDECYT, Chile N° 11180197, ANID, Chile - provided to Paula Celis - Plá.

## Acknowledgments

The authors thank the Mass Spectrometry and the Photobiology Units, Central Research Support Services (SCAI), University of Málaga, Spain, as well as General Direction Research, University of Playa Ancha - Chile.

## Conflict of interest

The authors declare that the research was conducted in the absence of any commercial or financial relationships that could be construed as a potential conflict of interest.

## Publisher’s note

All claims expressed in this article are solely those of the authors and do not necessarily represent those of their affiliated organizations, or those of the publisher, the editors and the reviewers. Any product that may be evaluated in this article, or claim that may be made by its manufacturer, is not guaranteed or endorsed by the publisher.

## References

[B1] Abdala-Díaz R. Cabello-Pasini A. Márquez-Garrido E. López F. (2014). Intra-thallus variation of phenolic compounds, antioxidant activity, and phenolsulphatase activity in *Cystoseira tamariscifolia* (Phaeophyceae) from southern Spain. Cienc. Mar. 40, 1–10. doi: 10.7773/cm.v40i1.2350

[B2] Adams W.W.III. Zarter C. R. Mueh K. E. Amiard V. S. E. Demmig-Adams B. (2006). “Energy dissipation and photoinhibition: a continuum of photoprotection,” in Photoprotection, photoinhibition, gene regulation, and environment. Eds. Demmig-Adams B. Adams W.W.III. Mattoo A. (Dordrecht, the Netherlands: Springer), 49–64.

[B3] Álvarez - Gómez F. Bouzon Z. Korbee N. Celis-Plá P. S. M. Schmidt E. C. Figueroa F. L. (2017). Combined effects of UVR and nutrients on cell ultrastructure, photosynthesis, and biochemistry in *Gracilariopsis longissima* (Gracilariales, rhodophyta). Algal. Res. 26, 190–202. doi: 10.1016/j.algal.2017.07.022

[B4] Anderson M. J. Gorley R. N. Clarke K. R. (2008). “PERMANOVA+ for *PRIMER: Guide to software and statistical methods* ,” (Plymouth, UK: PRIMER-E).

[B5] Bilger W. Björkman O. (1990). Role of the xanthophyll cycle in photoprotection elucidated by measurements of light-induced absorbance changes, fluorescence, and photosynthesis in leaves of *Hedera canariensis* . Photosynth. Res. 25, 173–185. doi: 10.1007/BF00033159 24420348

[B6] Bischof K. Gómez I. Molis M. Hanelt D. Karsten U. Lüder U. . (2006). Ultraviolet radiation shapes seaweed communities. Rev. Environ. Sci. Biot. 5, 141–166. doi: 10.1007/s11157-006-0002-3

[B7] Blois M. S. (1958). Antioxidant determinations by the use of a stable free radical. Nature. 181, 1199–1200. doi: 10.1038/1811199a0

[B8] Buschmann A. H. Pereda S. V. Varela D. A. Rodríguez-Maulén J. López A. González-Carvajal L. . (2014). Ecophysiological plasticity of annual populations of giant kelp (*Macrocystis pyrifera*) in a seasonally variable coastal environment in the northern Patagonian inner seas of southern Chile. J. Appl. Phycol. 26, 837–847. doi: 10.1007/s10811-013-0070-z

[B9] Cabello-Pasini A. Aguirre-von-Wobeserb E. Figueroa F. L. (2000). Photoinhibition of photosynthesis in *Macrocystis pyrifera* (Phaeophyceae), *Chondrus crispus* (Rhodophyceae) and *Ulva lactuca* (Chlorophyceae) in outdoor culture systems. J. Photochem. Photobiol. B: Biol. 57, 169–178. doi: 10.1016/S1011-1344(00)00095-6 11154083

[B10] Cabrera S. Ipiña A. Damiani A. Cordero R. R. Piacentini R. D. (2012). UV Index values and trends in Santiago, Chile (33.5°S) based on ground and satellite data. J. Photochem. Photobiol. B. 115, 73–84. doi: 10.1016/j.jphotobiol.2012.06.013 22883148

[B11] Celis-Plá P. S. M. Bouzon Z. Hall-Spencer J. M. Schmidt E. Korbee N. Figueroa F. L. (2016). Seasonal biochemical and photophysiological responses in the intertidal macroalga *Cystoseira tamariscifolia* (Ochrophyta). Mar. Environ. Res. 115, 89–97. doi: 10.1016/j.marenvres.2015.11.014 26724873

[B12] Celis-Plá P. S. M. Hall-Spencer J. M. Antunes-Horta P. Milazzo M. Korbee N. Cornwall C. E. . (2015). Macroalgal responses to ocean acidification depend on nutrient and light levels. Front. Mar. Sci. 2, 26. doi: 10.3389/fmars.2015.00026

[B13] Celis-Plá P. S. M. Kappes J. L. Figueroa F. L. Pereda S. Villegas K. Altamirano R. . (2021). Solar radiation as an isolated environmental factor in an experimental mesocosm approach for studying photosynthetic acclimation of *Macrocystis pyrifera* (Ochrophyta). Front. Plant Sci. 12, 622150. doi: 10.3389/fpls.2021.622150 34276713 PMC8283697

[B14] Celis-Plá P. S. M. Korbee N. Gómez-Garreta A. Figueroa F. L. (2014). Seasonal photoacclimation patterns in the intertidal macroalga *Cystoseira tamariscifolia* (Ochrophyta). Sci. Mar. 78 (3), 377–388. doi: 10.3989/scimar.04053.05A

[B15] Celis-Plá P. S. M. Martínez B. Korbee N. Hall-Spencer J. M. Figueroa F. L. (2017). Photoprotective responses in a brown macroalgae. To increases in CO_2_ and temperature. Mar. Environ. Res. 130, 157–165. doi: 10.1016/j.marenvres.2017.07.015 28764959

[B16] Demmig-Adams B. Adams W. W. 3rd (2006). Photoprotection in an ecological context: the remarkable complexity of thermal dissipation. New. Phytol. 172, 11.e21. doi: 10.1111/j.1469-8137.2006.01835.x 16945085

[B17] Eilers P. H. C. Peeters J. C. H. (1988). A model for the relationship between light intensity and the rate of photosynthesis in phytoplankton. Ecol. Model. 42, 199–215. doi: 10.1016/0304-3800(88)90057-9

[B18] Falkenberg L. J. Connell S. D. Russell B. D. (2013). Disrupting the effects of synergies between stressors: improved water quality dampens the effects of future CO_2_ on a marine habitat. J. Appl. Ecol. 50, 51–58. doi: 10.1111/1365-2664.12019

[B19] Figueroa F. L. Bonomi-Barufi J. Celis-Plá P. S. M. Nitschke U. Arenas F. Connan. S. . (2021). Short-term effects of increasing CO_2_, nitrate, and temperature on photosynthetic activity in *Ulva rigida* (Chlorophyta) estimated by different pulse amplitude modulated fluorometers and oxygen evolution. J. Exp. Bot. 72, 491–509. doi: 10.1093/jxb/eraa473 33064811

[B20] Figueroa F. L. Celis-Plá P. S. M. Martínez B. Korbee N. Trilla A. Arenas F. (2019). Yield losses and electron transport rate as indicators of thermal stress in *Fucus serratus* (Ochrophyta). Algal. Res. 41, 101560. doi: 10.1016/j.algal.2019.101560

[B21] Figueroa F. L. Conde-Álvarez R. Gómez I. (2003). Relations between electron transport rates determined by pulse amplitude modulated chlorophyll fluorescence and oxygen evolution in macroalgae under different light conditions. Photosynth. Res. 75, 259–275. doi: 10.1023/A:1023936313544 16228606

[B22] Figueroa F. L. Domínguez-González B. Korbee N. (2014). Vulnerability and acclimation to increased UVB in the three intertidal macroalgae of different morpho-functional groups. Mar. Env. Res. 101, 8–21. doi: 10.1016/j.marenvres.2014.01.009 24556033

[B23] Gilmore A. M. (1997). Mechanistic aspects of xanthophyll cycle-dependent photoprotection in higher plant chloroplast and leaves. Physiol. Plant 99 (1), 197–209. doi: 10.1111/j.1399-3054.1997.tb03449.x

[B24] Gómez I. Español S. Véliz K. Huovinen P. (2016). Spatial distribution of phlorotannins and its relationship with photosynthetic UV tolerance and allocation of storage carbohydrates in blades of the kelp *Lessonia spicata* . Mar. Biol. 163 (5), 110. doi: 10.1007/s00227-016-2891-1

[B25] Hanelt D. Figueroa F. L. (2012). “Physiological and photomorphogenic effects of light of marine macrophytes,” in Seaweed biology ecological studies. Eds. Wienke C. Bischof K. (Berlin Heidelberg: Springer-Verlag), 3–23.

[B26] Heinrich S. Valentin K. Frickenhaus S. John U. Wiencke C. (2012). Transcriptomic analysis of acclimation to temperature and light stress in(Phaeophyceae). PloS One 7 (8), e44342. doi: 10.1371/journal.pone.0044342 22937172 PMC3429442

[B27] Hemat R. A. S. (2007) (Dublin, Ireland), 83–85.

[B28] Huovinen P. Gómez I. (2006). A five-year study of solar ultraviolet radiation in southern Chile (39°S): Potential impact on physiology of coastal marine algae? Photochem. Photobiol. 82, 515–522, (20. doi: 10.1562/2005-07-05-RA-601 16613507

[B29] Lu Y. Wang H. Wang Q. Zhang Y. Yu Y. Qian Y. (2017). Global anthropogenic heat emissions from energy consumption. Clim. Change 145 (3-4), 459–468. doi: 10.1007/s10584-017-2092-z

[B30] Moenne A. González A. Sáez C. A. (2016). Mechanisms of metal tolerance in marine macroalgae, with emphasis on copper tolerance in Chlorophyta and Rhodophyta. Aquat. Toxicol 176, 30–37. 27107242 10.1016/j.aquatox.2016.04.015

[B31] Murata N. Takahashi S. Nishiyama Y. Allakhverdiev S. I. (2007). Photoinhibition of photosystem II under environmental stress. Biochim. Biophys. Acta 1767 (6), 414–421. doi: 10.1016/j.bbabio.2006.11.019 17207454

[B32] Palacios M. Osman D. Ramírez J. Huovinen P. Gómez I. (2021). Photobiology of the giant kelp *Macrocystis pyrifera* in the land terminating glacier fjord yendegaia (Tierra del fuego): A look into the future? Sci. Total Environ. 751, 141810. doi: 10.1016/j.scitotenv.2020.141810 32882566

[B33] Parsons T. R. Strickland J. D. H. (1963). Discusson of spectrophometric determination of marine-plant pigments, with revised equations for ascertaining chlorophyll-a and carotenois. ICES J. Mar. Sci. 21, 105–156. doi: 10.1016/0011-7471(65)90662-5

[B34] Pérez-lloréns J. L. Vergara J. J. Olivé I. Mercado J. M. Conde-Álvarez R. (2014). “Autochthonous seagrasses,” in The Mediterranean Sea: Its history and present challenges. Eds. Goffredo S. Dubinsky Z. (Netherlands, Dordrecht: Springer), 137–158.

[B35] Quintano E. Celis-Plá P. S. M. Martínez B. Díez I. Muguerza N. Figueroa F. L. . (2019). Ecophysiological responses of threatened red algae to increased irradiance in an *in-situ* transplant experiment. Mar. Env. Res. 144, 166–177. doi: 10.1016/j.marenvres.2019.01.008 30683559

[B36] Raven J. A. (2011). The cost of photoinhibition. Physiol. Plant 142, 87–104. doi: 10.1111/j.1399-3054.2011.01465.x 21382037

[B37] Raven J. A. Hurd C. L. (2012). Ecophysiology of photosynthesis in macroalgae. Photosynth. Res. 113, 105e125. doi: 10.1007/s11120-012-9768-z 22843100

[B38] Ritchie R. J. . (2006). Consistent sets of spectrophotometric chlorophyll equations for acetone, methanol and ethanol solvents Photosynthesis research 89, 1, 27–414:28 PM 9/19/2022. 16763878 10.1007/s11120-006-9065-9

[B39] Sáez C. A. González A. Contreras R. A. Moody A. J. Moenne A. Brown M. T. (2015). A novel field transplantation technique reveals intra-specific metal-induced oxidative responses in strains of *Ectocarpus siliculosus* with different pollution histories. Environ. pollut. 199, 130–138. doi: 10.1016/j.envpol.2015.01.026 25645062

[B40] Sathya R. Kanaga N. Sankar P. Jeeva S. (2017). Antioxidant properties of phlorotannins from brown seaweed *Cystoseira trinodis* (Forsskal). C. Agardh. Arab. J. Chem. 10, S2608–S2614. doi: 10.1016/j.arabjc.2013.09.039

[B41] Schreiber U. Endo T. Mi H. Asada K. (1995). Quenching analysis of chlorophyll fluorescence by the saturation pulse method: particular aspects relating to the study of eukaryotic algae and cyanobacteria. Plant Cell. Physiol. 36, 873–882. doi: 10.1093/oxfordjournals.pcp.a078833

[B42] Underwood T. (1997). Experiments in ecology. their logical design and interpretation using analysis of variance (Cambridge UK: Cambridge University Press).

[B43] Varela D. A. Henriquez L. A. Fernández P. Leal P. Hernández-González M. C. Figueroa F. L. . (2018). Photosynthesis and nitrogen uptake of the giant kelp *Macrocystis pyrifera* grown close to salmon farms. Mar. Environ. Res. 135, 93–102. doi: 10.1016/j.marenvres.2018.02.002 29428528

[B44] Wiencke C. Gómez I. Pakker H. Flores-Moya A. Altamirano M. Hanelt D. . (2000). Impact of UV-radiation on viability, photosynthetic characteristics, and DNA of brown algal zoospores: implications for depth zonation. Mar. Ecol. Prog. Ser. 197, 217–229. doi: 10.3354/meps197217

[B45] Zúñiga A. Sáez C. A. Trabal A. Figueroa F. L. Pardo D. Navarrete C. . (2021). Seasonal photoacclimation and vulnerability patterns in the brown macroalga Lessonia spicata (Ochrophyta). Water 13, 1, 6. doi: 10.3390/w13010006

