# Supplementary material for: Daily changes on seasonal ecophysiological responses of the intertidal brown macroalga Lessonia spicata: Implications of climate change
Source: Front Plant Sci. 2022 Sep 28;13:941061. doi: 10.3389/fpls.2022.941061 (PMC9554264; doi:10.3389/fpls.2022.941061)
Supplement: Supplementary file 1 [file Table_1.docx]

**SUPPLEMENTARY MATERIAL**

**Table S1**. ANOVA results for ecophysiological responses of *Lessonia spicata* (*p* < 0.05). Results considered were *F_v_/F_m_*, ETR_max_, Carbon, Nitrogen, Chlorophyll *a*, Chlorophyll *c*, Carotenoids, phenolic compounds, DPPH, H_2_O_2_, and TBARS, under daily cycle experiments during Autumn, Winter, Spring, and Summer between 2019 and 2020 in Valparaíso Bay.

|  |  |  | ***Lessonia spicata*** | | |
| --- | --- | --- | --- | --- | --- |
|  |  | ***df*** | ***MS*** | ***F*** | ***P*** |
| ***Fv/Fm*** | *Season (s)* | 3 | 0.0006 | 1.9 |  |
|  | *Hour (h)* | 4 | **0.0154** | **44.1** | ****** |
|  | *s*h* | 12 | **0.0078** | **2.2** | ****** |
|  | *Res* | 100 | **0.0035** |  |  |
| **ETR_max_** | *Season (s)* | 3 | **15366.4** | **112.2** | ****** |
|  | *Hour (h)* | 4 | **3126.1** | **22.8** | ****** |
|  | *s*h* | 12 | **1342.4** | **10.0** | ****** |
|  | *Res* | 100 | 137.1 |  |  |
| **NPQ_max_** | *Season (s)* | 3 | **1.301** | **14.6** | ****** |
|  | *Hour (h)* | 4 | **1.401** | **15.8** | ****** |
|  | *s*h* | 12 | **1.201** | **14.2** | ****** |
|  | *Res* | 100 | 0.865 |  |  |
| **Carbon** | *Season (s)* | 3 | **547** | **5.31** | ****** |
|  | *Hour (h)* | 2 | 11 | 0.10 |  |
|  | *s*h* | 6 | **177** | **1.72** | ****** |
|  | *Res* | 24 | **103** |  |  |
| **Nitrogen** | *Season (s)* | 3 | **67.30** | **55.64** | ****** |
|  | *Hour (h)* | 2 | **4.88** | **4.03** | ****** |
|  | *s*h* | 6 | **2.14** | **1.77** | ****** |
|  | *Res* | 24 | **1.21** |  |  |
| **Chlorophyll *a*** | *Season (s)* | 3 | **0.287** | **28.952** | ****** |
|  | *Hour (h)* | 2 | 0.019 | 1.983 |  |
|  | *s*h* | 6 | 0.005 | 0.509 |  |
|  | *Res* | 24 | 0.009 |  |  |
| **Chlorophyll *c*** | *Season (s)* | 3 | **0.001** | **12.782** | ****** |
|  | *hour (h)* | 2 | **0.002** | **14.689** | ****** |
|  | *s*h* | 6 | **0.001** | **3.796** | ****** |
|  | *Res* | 24 | 0.001 |  |  |
| **Carotenoids** | *Season (s)* | 3 | **0.126** | **92.170** | ****** |
|  | *hour (h)* | 2 | 0.009 | 0.710 |  |
|  | *s*h* | 6 | **0.011** | **8.480** | ****** |
|  | *Res* | 24 | 0.001 |  |  |
| **Phenolic Compounds** | *Season (s)* | 3 | **145.723** | **184.197** | ****** |
|  | *Hour (h)* | 2 | **25.110** | **31.746** | ****** |
|  | *s*h* | 6 | **11.041** | **13.957** | ****** |
|  | *Res* | 24 | 0.791 |  |  |
| **DPPH** | *Season (s)* | 3 | **88.6** | **17.70** | ****** |
|  | *Hour (h)* | 2 | **43.5** | **8.69** | ****** |
|  | *s*h* | 6 | **58.7** | **11.73** | ****** |
|  | *Res* | 24 | 5.0 |  |  |
| **H_2_O_2_** | *Season (s)* | 3 | **0.313** | **90.404** | ****** |
|  | *Hour (h)* | 2 | **0.030** | **8.664** | ****** |
|  | *s*h* | 6 | 0.002 | **0.734** | ****** |
|  | *Res* | 24 | 0.003 |  |  |
| **TBARS** | *Season (s)* | 3 | **10.305** | **64.180** | ****** |
|  | *Hour (h)* | 2 | **1.012** | **6.304** | ****** |
|  | *s*h* | 6 | 0.625 | **3.891** | ****** |
|  | *Res* | 24 | 0.161 |  |  |

*Res: Residual*
